# Supplementary material for: Hypoxia Affects the Antioxidant Activity of Glutaredoxin 3 in Scylla paramamosain through Hypoxia Response Elements
Source: Antioxidants (Basel). 2022 Dec 29;12(1):76. doi: 10.3390/antiox12010076 (PMC9855028; doi:10.3390/antiox12010076)
Supplement: Supplementary file 1 [file antioxidants-12-00076-s001.zip › Supplementary Materials-Table S1.pdf]

Table S1 Sequences of primers used in this study.

Primer name	Primer Sequence (5’- 3’)
RACE	
SpGrx3-3′ RACE-1	ATTCATGAAGGGAGATCGGGAAGCA
SpGrx3-3′ RACE-2	TCAGCAACTGGCCAACTTATCCAC
SpGrx3-5′ RACE-1	TGCAGCAGACCTCACCAATAAAGTG
SpGrx3-5′ RACE-2	GTATTGTGCCAGCTGAAGACCTGTCA
Genome walking	
GSP-R1	TGCGCGGTGTAGTGTTAGTGAT
GSP-R2	AGAGCACCAATAGCAACAGCAG
GSP-R3	AGGCTGTTGAAGAAAGCCTACTTG
Real-time PCR	
RT-*SpGrx3*-F	ATGGAGAACTGGTTGGCGGG
RT-*SpGrx3*-R	GGTGCTTCCCGATCTCCCTT
RT-Grx2-F	GCTGCAGTTGACATGGAAGGT
RT-Grx2-R	CCACCAACAAACACTCGAGGAA
RT-GST-F	CACAGCCCATTCGCCTCC
RT-GST-R	GGTCATACTTGCGTCCCAGATAG
RT-GPx-F	TCTGTTACCGCTGGTGGCTG
RT-GPx-R	TGTGAACCGTTTAGTGTTTTGGC
18S rRNA-F	GAGAAACGGCTACCACATCC
18S rRNA-R	GATACGCTCATTCCGATTACAG

ORF amplification	
SpGrx3-pAc5.1-F	tagtccagtgtggtggaattcATGTCTGTTACTAAGGTTGC<br>CACTGA
SpGrx3-pAc5.1-R	gaagggccctctagactcgagCTCTCCCTTCAAGGTTCCC<br>ATT
RNA interference	
ds*SpGrx3*-F	GGATCCTAATACGACTCACTATA<br>GGGCAGTCCCCACATTTCTGTT
ds*SpGrx3*-R	GGATCCTAATACGACTCACTATA<br>GGACGGAAGCCCAGTATCATTG
dsGFP-F	GGATCCTAATACGACTCACTATAGGG<br>TGGAGTGGTCCCAGTTCTTGTTGA
dsGFP-R	GGATCCTAATACGACTCACTATAGGG<br>GCCATTCTTTGGTTTGTCTCCCAT
Dual-luciferase reporter assays	
pGL3-*SpGrx3*-F1	gcgtgctagcccgggctcgag<br>CTGTTCCCTAGCGGCAGTCA
pGL3-*SpGrx3*-R1.1	cagtaccggaatgccaagctt<br>TTCAAACAGTTCAGATTTTTTTATAAAA
pGL3-*SpGrx3*-F2	gcgtgctagcccgggctcgag<br>ACTGAGTAAGTTAAGAAGTTATTGGAAAATT
pGL3-*SpGrx3*-F3	gcgtgctagcccgggctcgag

	GAAACAAAATCTCAAATATACGAAACAA
pGL3-*SpGrx3*-R	cagtaccggaatgccaagcttGAGAAGGAAGCGGCGGTG
SpGrx3-F3.1-F	GGACACtatacaACTGCTGTTGCTATTGGTGCTCT
SpGrx3-F3.1-R	AGCAGTtgtataGTGTCCTCAGTGGCAGCCG
